# Roma Ethnicity and Sex-Specific Associations of Serum Uric Acid with Cardiometabolic and Hepatorenal Health Factors in Eastern Slovakian Population: The HepaMeta Study

**DOI:** 10.3390/ijerph17207673

**Published:** 2020-10-21

**Authors:** Maria Pallayova, Marek Brenisin, Alina Putrya, Martin Vrsko, Sylvia Drazilova, Martin Janicko, Maria Marekova, Daniel Pella, Andrea Madarasova Geckova, Peter Urdzik, Peter Jarcuska, HepaMeta Team

**Affiliations:** 1 Department of Medical Physiology, Faculty of Medicine, Pavol Jozef Safarik University, 04011 Kosice, Slovakia; maria.pallayova@upjs.sk; 2 Department of Pathological Physiology, Faculty of Medicine, Pavol Jozef Safarik University, 04011 Kosice, Slovakia; marek.brenisin@upjs.sk; 3 2nd Department of Cardiology, Faculty of Medicine, Pavol Jozef Safarik University, 04011 Kosice, Slovakia; aliputrya@gmail.com (A.P.); daniel.pella@upjs.sk (D.P.); 4 Institute of Cardiovascular Diseases, Faculty of Medicine, Pavol Jozef Safarik University, 04011 Kosice, Slovakia; 5 Department of Internal Medicine, AGEL Hospital, Kosice-Saca, 04015 Kosice, Slovakia; vrskomartin@gmail.com; 6 2nd Department of Internal Medicine, Faculty of Medicine, Pavol Jozef Safarik University, 04011 Kosice, Slovakia; drazilovasylvia@gmail.com (S.D.); martin.janicko1@upjs.sk (M.J.); peter.jarcuska@upjs.sk (P.J.); 7 Louis Pasteur University Hospital, 04011 Kosice, Slovakia; 8 Department of Medical and Clinical Biochemistry, Faculty of Medicine, Pavol Jozef Safarik University, 04011 Kosice, Slovakia; maria.marekova@upjs.sk; 9 Department of Health Psychology, Faculty of Medicine, Pavol Jozef Safarik University, 04011 Kosice, Slovakia; andrea.geckova@upjs.sk; 10 Department of Gynaecology and Obstetrics, Faculty of Medicine, Pavol Jozef Safarik University, 04011 Kosice, Slovakia

**Keywords:** C-reactive protein, health status, Roma, sex, uric acid

## Abstract

*Background:* Health characteristics associated with uric acid (UA) in the Roma minority remain less well known. The study sought to determine the ethnicity- and sex-specific associations of serum UA with health factors in Eastern Slovakian Roma and non-Roma populations. *Methods:* Data from the comparative cross-sectional HepaMeta study conducted in Slovakia in 2011 were used. The study enrolled 452 Roma subjects (35.2% men) and 403 non-Roma individuals (45.9% men) aged 18–55 years. *Results:* All study parameters differed between the sexes in both the Roma and non-Roma participants (*p* < 0.05). UA was related to sex with odds ratio for female sex 0.873, 95% CI 0.853–0.893 (*p* < 0.0001) per 10-unit increase of UA. Average level of UA ± standard deviation was lower in Roma than in non-Roma (226.54 ± 79.8 vs. 259.11 ± 84.53 umol/L; *p* < 0.0001). The Roma population presented with greater levels of high-sensitivity C-reactive protein (hsCRP) (3.07 ± 4 mg/L vs. 1.98 ± 2.83 mg/L; *p* < 0.0001) and ferritin in Roma males (403.78 ± 391.84 vs. 302.67 ± 236.26 mg/L; *p* < 0.0001). *Conclusions:* Serum UA is sex- and ethnicity specific. Elevated levels of hsCRP and ferritin particularly in Roma males can reflect low-grade systemic inflammation and thus serve as a marker of an increased cardiovascular risk.

## 1. Introduction

Cardiometabolic diseases belong to the most frequent disorders treated worldwide and now emerge as a public health priority. They are associated with a significantly increased morbidity, overall disability, and premature mortality. Evidence suggests that cardiometabolic diseases are more prevalent among ethnic minorities [1,2,3].

The Roma population is one of the major and oldest ethnic minorities in Europe and the second-largest minority group in the Slovak Republic. The majority of the Roma people live in the eastern and southern parts of Slovakia. An increasing body of evidence suggests that the Roma population is at increased cardiovascular and metabolic risk [2,4,5]. Yet, the literature on novel biomarkers of cardiometabolic diseases in this population is scarce [2]. A high burden of noncommunicable cardiovascular and metabolic diseases in the Roma population highlights the need for further investigations of novel biomarkers that would facilitate the diagnosis and would also help develop culturally sensitive health literacy interventions and prevention programs among the vulnerable Roma patient groups.

Serum uric acid is the main final enzymatic end product of the common pathway of purine metabolism in humans [6]. Elevated serum uric acid is a marker of chronic inflammation and has been suggested to be associated with metabolic syndrome [7], non-alcoholic fatty liver disease (NAFLD) [7], and a range of inflammatory markers [8]. In addition to human studies, recent animal model evidence strongly suggests that uric acid is directly causal for fatty liver and metabolic syndrome phenotypes [9]. On the other hand, uric acid is an important physiological antioxidant. Serum uric acid accounts for about 50% of extracellular antioxidant activity, suggesting that hyperuricemia may have a protective role in diseases characterized by high levels of oxidative stress [10]. 

Inflammation is a well-established obligatory marker of the initiation and the progression of atherosclerosis [11]. The increasing need for the rapid and effective evaluation of cardiovascular risk associated with inflammation has led to the wide use of high-sensitivity C-reactive protein (hsCRP), which is a member of the short pentraxin family working as an acute marker and as an event predictor [11]. The expression of this circulating biomarker is being stimulated by proinflammatory cytokines, e.g., interleukin-6 (IL-6), IL-1β, tumor necrosis factor-α (TNF-α), and others [12]. High-sensitivity CRP can be used to not only identify a high-risk group for recurrent events in patients with manifest atherosclerosis, but the levels of hsCRP might also further aid in tailoring risk-guided statin treatment [13].

The assessment of renal function is essential to manage and follow up the patients with metabolic syndrome. The current evidence indicates that there is a higher prevalence of metabolic syndrome in the Roma population [2]. Moreover, previous studies have suggested that Roma compared to non-Roma have a 2.85 times higher risk of prevalence of end-stage renal disease [14], and Roma females have half-higher odds for nephropathy than non-Roma females [15]. While serum creatinine is routinely used for the assessment of renal function, a creatinine-based estimated glomerular filtration rate does not always reflect true renal function because of possible muscle wasting and/or impaired liver function. By contrast, cystatin C, one of the most common surrogate markers of glomerular filtration rate, is unrelated to muscle volume and liver function. In addition, cystatin C is not influenced by sex, ethnicity, sarcopenia, and liver diseases [16].

At present, the health characteristics of the Roma minority within the Slovak Republic and their associations with uric acid have not been systematically studied and remain less well known. Therefore, we examined a Roma population for some anthropometrical and biochemical parameters. In previous studies, we have demonstrated that Roma compared to non-Roma had higher rates of some cardiovascular disease risk factors (smoking, obesity, low HDL cholesterol) [17], metabolic syndrome [2,5], a lack of physical activity [18], as well as unhealthy eating habits [19]. The primary aim of the present study was to determine a comprehensive biochemical profile and ethnicity-specific associations of serum uric acid with cardiometabolic and hepatorenal health factors in Eastern Slovakian Roma and non-Roma populations. Secondary goals were to describe the sex-specific associations of serum uric acid with health factors in these populations.

## 2. Materials and Methods

### 2.1. Study Design and Setting

Cross-sectional population-based study that included participants from segregated Roma settlements and participants from the majority population. 

Ethics Committee of the Faculty of Medicine at Pavol Jozef Šafárik University in Košice approved the study (the project identification code: Protocol number 104/2011) on 12 January 2011.

### 2.2. Subjects and Measures

A detailed description of the methods used was published by Madarasova-Geckova et al. (2014) [20].

- The target population participants recruitment: Nineteen general practitioners were randomly chosen from a list of general practitioners in the catchment area with Roma population in the Košice region, and 12 general practitioners agreed to participate. Roma were recruited by local Roma community workers from the selected settlements under unpredictable conditions (unknown response rate). From all Roma in the settlements who received information about the study, 452 have opted to participate.
- The majority population participants recruitment: Non-Roma participants were randomly selected via a two-stage process. Seven general practitioners were randomly chosen from a list of general practitioners in the catchment area without Roma population in the Košice region, and five agreed to take part in the study (380 subjects were randomly selected from the majority population). Nineteen general practitioners were randomly chosen from a list of general practitioners in the catchment area with Roma population in the Košice region, and 12 general practitioners agreed to participate (330 subjects were randomly selected from the majority population). Therefore, a total of 710 subjects from the majority population were randomly chosen from a list of patients. These were contacted via phone and via e-mail by trained research assistants, who provided the information about the study and invited them to participate. A total of 403 non-Roma were recruited and participated in the study (response rate of 56.8%).
  1. Questionnaire—medical history—records from the General practitioners office
  2. Anthropometric measures and blood sample analyses after an overnight fasting.
  3. Clinical Biochemistry tests for determination substrates: cystatin C (cysC), creatinine, minerals—serum iron level (Fe), proteins (ferritin and high sensitivity C-reactive protein (hs-CRP) as risk factor), enzymes (alanine-aminotransferase—ALT, aspartate-aminotransferase—AST, gamma-glutamyl transferase—GGT or GMT) glucose, uric acid (UA). All biochemical parameters were determined by routine biochemical methods on an analyzer ADVIA 2400 or 1650. Ferritin was measured by chemiluminescent immunoassay (CLIA) on an analyzer ADVIA Centaur (Siemens).

### 2.3. Statistical Analysis

Categorical data are presented as absolute and relative counts. Interval data are presented as mean ± standard deviation. Comparisons of distributions among multiple groups were performed by ANOVA with LSD post-hoc analysis. A comparison of interval variables between two groups has been performed by the Student t-test and categorical variables have been performed by the chi-squared test. The relationship between uric acid and dependent variables was explored by linear regression adjusted for age and sex if categorized by ethnicity, or age and ethnicity if categorized by sex for interval dependent variables and logistic regression for categorical dependent variables. Regression coefficients are reported multiplied by 10 because of very small effect sizes due to large distribution width. These analyses were performed on the whole cohort and also on subgroups based on ethnicity and sex. A comparison of regression curves between Roma and non-Roma groups was performed by fitting a linear regression model with uric acid, age, sex, ethnicity category, and interaction term (ethnicity category*uric acid) as predictors. Similarly, the comparison was performed between males and females. Statistical analyses were performed in SPSS statistical package (version 22, IBM Corp., New York, NY, USA).

## 3. Results

The study sample comprised 855 enrolled participants, of which 511 (59.8%) were females. The Roma subgroup consisted of 452 subjects (35.2% men) recruited from segregated settlements, while the non-Roma subgroup consisted of 403 individuals (45.9% men). Participants were excluded per analysis on the ground of missing data (number of included participants per analysis is indicated in the tables). The subjects were aged 18–55 years. The average age ± standard deviation was 34.1 ± 8.4 years (Roma 34.7 ± 9.1 years; non-Roma 33.5 ± 7.3 years). A summary of the study parameters broken down into four groups based on sex and ethnicity is presented in Table 1. Uric acid, ferritin, and hsCRP were significantly lower in non-Roma males compared to their Roma counterparts. Non-Roma females had significantly lower body mass index (BMI), cystatin C, ferritin, and hsCRP, but higher uric acid, creatinine and serum iron compared to their Roma counterparts. A higher proportion of Roma were poor and smokers compared to non-Roma; no difference in proportion of significant alcohol drinkers was observed. Uric acid ranged from 24.4 to 585.6 micromol/L, with fairly symmetrical distribution (skewness 0.599) approaching a bell curve (kurtosis 0.308); however it was not normal (Shapiro–Wilk < 0.0001). Levels of uric acid in the Roma population were significantly lower than in non-Roma (226.54 ± 79.8 vs. 259.11 ± 84.53 umol/L; *p* < 0.001).

In logistic regression adjusted for age, uric acid was significantly related to sex with odds ratio (OR) for female sex 0.873, 95% CI 0.853–0.893; *p* < 0.0001 per 10-unit increase of uric acid. Table 2 presents the results of linear regression analyses with the uric acid adjusted for age and sex as the only predictor in all analyses. The results showed a significant relationship between uric acid and almost all the analyzed dependent variables except for ALT. However, the observed effect sizes were very small. The largest effect sizes were observed for BMI (0.202 kg/m^2^ rise per 10 micromol/L rise of uric acid), creatinine (0.326 micromol/L rise per 10 micromol/L rise of uric acid), and hsCRP (0.09 mg/L rise per 10 unit rise of uric acid).

A separate set of analyses was conducted after adjustment for BMI in addition to age and sex (Table S1). After adjustment for BMI, only systolic and diastolic blood pressures were no longer associated with uric acid, while all other significant associations reported in Table 2 remained.

### 3.1. Subgroup Analyses

To further explore the ethnicity- and sex-specific associations of serum uric acid, the bivariate regression analyses were performed for Roma and non-Roma subgroups and for males and females separately. 

#### 3.1.1. Roma 

Uric acid in the Roma subgroup ranged from 24.4 to 538.19 micromol/L. In logistic regression adjusted for age, uric acid was significantly related to sex with OR for female sex 0.893, 95% CI 0.867–0.920; *p* < 0.0001 per 10-unit increase of uric acid. Table 3 presents the results of linear regression analyses for the Roma subgroup with the uric acid adjusted for age and sex as the only predictor in all analyses. In the Roma subgroup, significant linear associations of uric acid levels were observed with BMI, age, systolic and diastolic blood pressure, albumin, creatinine, cystatin C, ferritin, and hsCRP. The strongest associations were with BMI (0.289 kg/m^2^ rise per 10 micromol/L rise of uric acid) and with hsCRP (10-unit rise of uric acid reflected into 0.125 unit rise of hsCRP). 

#### 3.1.2. Non-Roma

Uric acid in the non-Roma subgroup ranged from 74.6 to 585.6 micromol/L. In logistic regression adjusted for age, uric acid was significantly related to sex with OR for female sex 0.849, 95% CI 0.818–0.881; *p* < 0.0001 per 10-unit increase of uric acid. In the non-Roma subgroup (Table 4), significant linear associations of uric acid levels were observed with BMI, systolic and diastolic blood pressure, creatinine, cystatin C, ferritin, and hsCRP. The strongest association was with creatinine (10 units rise of uric acid reflected into 0.44 unit rise of creatinine) followed by BMI (0.164 kg/m^2^ rise per 10 micromol/L rise of uric acid).

#### 3.1.3. Roma vs. Non-Roma Comparison

In the Roma subgroup, compared to the non-Roma subgroup, we observed significant linear associations of uric acid with age and albumin, but not with the liver tests. In addition, smoking was associated with uric acid levels lower by 21 umol/L on average only in Roma. On the other hand, significant alcohol intake was associated with uric acid levels higher by 31 umol/L on average only in non-Roma. Serum iron was not associated with uric acid in any subgroup. All other parameters were significantly associated with uric acid in both subgroups. Table 5 shows the comparison of regression curves for variables significantly associated with uric acid in both subgroups. As shown in Table 5, the regression curve modeling associations between uric acid and BMI and between uric acid and hsCRP were significantly steeper in Roma compared to non-Roma population (differences between regression coefficients were statistically significant). No other difference in regression curves was observed between Roma and non-Roma subgroups.

#### 3.1.4. Males

Uric acid in males ranged from 58.19 to 585.57 micromol/L. Uric acid in males was significantly related to ethnicity with OR for non-Roma (adjusted for age) 1.059 (95% CI 1.032–1.088) per 10-unit increase of uric acid. Further associations of uric acid, adjusted for age and ethnicity, were observed with age (only adjusted for ethnicity), BMI, blood pressure, albumin, cystatin C, creatinine, iron, ferritin, and hsCRP (Table 6). The strongest associations in males were observed with creatinine (0.454 umol/L rise per 10 micromol/L rise of uric acid) and BMI (0.178 kg/m^2^ rise per 10 micromol/L rise of uric acid).

#### 3.1.5. Females

Uric acid in females ranged from 24.4 to 434.10 micromol/L. Uric acid in females was significantly related to ethnicity with OR for non-Roma (adjusted for age) 1.044 (95% CI 1.014–1.074) per 10-unit increase of uric acid. Further associations of uric acid, adjusted for age and ethnicity, were observed with BMI, blood pressure, AST, GMT, creatinine, ferritin, and hsCRP (Table 7). The strongest association of uric acid was observed with BMI (0.295 kg/m^2^ rise per 10 micromol/L rise of uric acid).

#### 3.1.6. Males vs. Females Comparison

Uric acid was significantly associated with age, iron, serum albumin, and cystatin C only in males. On the other hand, associations with AST and GMT were observed only in females. Uric acid was significantly associated with BMI, blood pressure, creatinine, ferritin, and hsCRP in both sexes. Smoking was associated with uric acid levels lower by 14.6 umol/L on average only in females. The slope of the regression curve modeling association between creatinine and uric acid was significantly steeper in males, and the slope of the curve modeling association between uric acid and hsCRP was significantly steeper in females (Table 8). No difference in the regression curve slope was observed between sexes for blood pressure, ferritin, or BMI.

## 4. Discussion

The primary finding of the present study is the comprehensive biochemical profile and ethnicity-specific associations of serum uric acid with cardiometabolic and hepatorenal health factors in Eastern Slovakian Roma and non-Roma populations. The secondary findings are sex-specific associations of serum uric acid with health factors in these populations. A high burden of noncommunicable cardiovascular and metabolic diseases in the Roma population highlights the need for the investigation of the novel biomarkers that has shown some remarkable associations between serum uric acid and cardiometabolic/hepatorenal pathophysiology.

Our study has demonstrated that serum uric acid is sex- and ethnicity-specific. The findings of lower uric acid levels in premenopausal females can be explained by the well-established uricosuric effects of estrogens [21]. Endogenous estradiol appears to decrease uric acid levels by lowering the post-secretory tubular reabsorption of uric acid [22]. Notably, estrogen may play a role in the regulation of expression or activity of uric acid transporters, specifically ABCG2 and SLC2A9; estrogen could mediate either direct transcriptional regulation of the transporter genes or activate transporter-specific transcription factors, including HNF4α [23]. Thus, using sex as a biological variable may provide key insights into understanding uric acid handling [24]. In our cohort, the serum uric acid levels were significantly decreased in the Roma population compared to non-Roma counterparts. These findings are in accordance with the previously reported observations [2,24] and could be attributed to a higher frequency of SLC22A12 variants causing renal hypouricemia 1 in the Roma population [24].

In the entire cohort, a significant relationship between uric acid and almost all analyzed dependent variables (hsCRP, creatinine, cystatin C, iron, ferritin, albumin, AST, GMT, BMI, and systolic and diastolic blood pressure) except ALT was observed with the largest effect sizes for BMI, creatinine, and hsCRP. Both in Roma and non-Roma populations, the serum uric acid level predicted BMI, systolic and diastolic blood pressure, creatinine, cystatin C, ferritin, and hsCRP. This substantiates previous findings in the literature demonstrating that hyperuricemia is strongly associated with metabolic syndrome, obesity, arterial hypertension, and other atherosclerosis-related conditions [7,25,26,27]. The associated inflammatory processes stimulate the expression of inflammatory biomarkers such as hsCRP and ferritin. In our study, significant associations of uric acid with hsCRP and ferritin were observed in the entire cohort as well as in both Roma and non-Roma populations. These values correlate favorably with Darmawan and colleagues [28] and further support the role of uric acid to stimulate inflammation through the production of p38 mitogen-activated protein kinases (MAPK), cyclooxygenase-2 (COX-2), and chemokinemonocyte chemoattractant protein-1 [28]. Furthermore, it has been reported that serum uric acid within the normal range correlated positively with interleukin-18 (IL-18), IL-6, and tumor necrosis factor-α (TNF-α) and also induced oxidative stress in adipocytes and vascular cells [28]. These mechanisms might help explain the observed relationships between the serum uric acid and hsCRP and ferritin. The remarkable results to emerge from the data are the positive associations of the serum uric acid with creatinine and cystatin C observed in the entire cohort as well as in both Roma and non-Roma populations. The kidneys play an essential role in the excretion of waste products and toxins such as urea, creatinine, and uric acid. Our findings concur well with the previous report of the significant positive correlation of serum uric acid with serum creatinine [29]. While the associations of uric acid levels with the progression of chronic kidney disease remain controversial [29,30], a meta-analysis published in 2018 showed that uric acid-lowering therapy may play an important role in delaying the progression of chronic kidney disease [31]. Remarkably, the positive association of uric acid with cystatin C in the absence of acute kidney injury in our study has further strengthened the hypothesis that the concomitant elevation of serum cystatin C and serum uric acid levels is associated with the presence of cardiometabolic risk accumulation [32,33].

The marked observations to emerge from the data comparison were additional significant positive linear associations of the serum uric acid with age and albumin in the Roma subgroup only. Of note, both uric acid and albumin are potent antioxidants and correlate with oxidative stress in some degree [34,35]. Uric acid can act as a peroxynitrite scavenger [36], while albumin can inhibit the production of free hydroxyl radicals [37] and scavenge peroxy radicals [38]. Based on the results, it can be assumed that Roma population may have an increased risk of oxidative stress. 

The comparison of the difference in regression curves between Roma and non-Roma subgroups showed significantly steeper regression curve modeling associations between uric acid and BMI and between uric acid and hsCRP in Roma compared to non-Roma. This finding indicates the presence of an increased low-grade systemic inflammation in Roma population compared to non-Roma counterparts most likely due to an increased cardiometabolic risk. These observations correlate well with previous findings [2,39] and further support the idea that the Roma are at increased risk for cardiovascular and metabolic diseases [4,5]. Other risk factors previously reported in Roma (smoking, obesity, lack of physical activity) could contribute to the low-grade systemic inflammation in the Roma population. Specifically, active smoking is a well-established traditional cardiovascular risk factor leading to smoking-induced oxidative stress as a trigger for a generalized vascular inflammation associated with cytokine release, the adhesion of inflammatory cells, and, ultimately, a disruption of endothelial integrity [40]. The source of inflammation in obese subjects is suggested to be mainly the visceral adipose tissue. Metabolic stress pathways in adipocytes induce inflammatory cascades accompanied by fibrotic processes and insulin resistance, along with progressive immune cell infiltration, the release of cytokines, and a subsequent spill-over of inflammation to the systemic circulation [41].

The Roma subgroup was predominantly female (*n* = 293; 64.8%) and slightly but significantly older (*p* = 0.043). The Roma people had significantly greater BMI and significantly lower levels of uric acid, creatinine, albumin, ALT, AST, GMT, and serum iron. By contrast, the serum level of hsCRP was significantly increased in Roma compared to non-Roma population. The finding of the increased BMI in the Roma people is in line with previous results demonstrating a higher prevalence of metabolic syndrome in the Roma population [2]. Interestingly, Roma females had significantly lower diastolic blood pressure and creatinine compared to non-Roma females. Low diastolic blood pressure is a marker of chronic disease and a marker of increased arterial stiffness [42]. Of importance is the reported J-shaped relation between diastolic blood pressure and mortality, where a diastolic blood pressure of 60 mmHg or less was associated with higher mortality [43]. Conditions (apart from the kidney disease) that might affect serum creatinine include extremes of muscle mass, nutrition, or physical activity status. Previous study has demonstrated unhealthy eating habits in the population living in Roma settlements most likely due to segregation or poverty and lower health literacy [19].

The secondary findings of the present study are considerable sex-specific associations of serum uric acid with health factors in Roma and non-Roma Eastern Slovakian populations. Compared to women in the entire cohort, men had significantly higher systolic blood pressure and higher levels of serum albumin, serum creatinine, cystatin C, liver enzymes, iron, and serum ferritin. While sex differences in serum creatinine, liver enzymes, iron, and serum ferritin have been widely reported, no sex differences have been noted for the serum albumin and cystatin C. Of note, both albumin and cystatin C levels, although higher in men than in women, were within the normal range. We cannot exclude possible other influences, e.g., the impact of nutritional factors, body composition, or the presence of a hidden cardiovascular risk. We did not investigate the presence of microalbuminuria, but based on clinical questionnaires and past medical history, the subjects did not suffer from any acute kidney injury. Importantly, the average levels of serum ferritin in men were elevated above the normal range. This finding was more pronounced in Roma males. Along with the significantly and clinically elevated average hsCRP, the increased serum ferritin in Roma males can reflect low-grade systemic inflammation and thus serve as a marker of subclinical atherosclerosis and predictor of coronary artery disease. Importantly, among the different serum markers considered, serum uric acid and ferritin are both associated with hepatic steatosis and metabolic syndrome and have emerged as possible predictors of severity of liver damage in NAFLD [44,45]. We did not evaluate NAFLD in our study, which precludes making inferences. Yet, systolic blood pressure was significantly increased in males (both Roma and non-Roma), which places them at increased cardiovascular risk.

To further explore the sex-specific associations of serum uric acid, the bivariate regression analyses were performed for males and females separately. Bivariate association analyses have demonstrated that uric acid was significantly associated with BMI, blood pressure, creatinine, ferritin, and hsCRP in both sexes independent of age and ethnicity. In our study, uric acid was significantly associated with age, iron, serum albumin, and cystatin C only in males. By contrast, the positive associations of uric acid with hepatic enzymes AST and GMT were observed only in females in our cohort. It is well established that elevated uric acid and hepatic markers are associated with metabolic risk. In addition, the association of lower uric acid with liver function (via lower AST and GMT) only in females could be explained by the previous studies that have reported a higher prevalence of NAFLD in men than in women; premenopausal women are equally protected from developing NAFLD as they are from cardiovascular disease [46]. Ultimately, the increasing serum uric acid levels in women over time may indicate the increasing risk of liver dysfunction with NAFLD development and its progression. The relationship between hepatic markers and uric acid in females in our cohort warrants further investigations. The slope of the regression curve modeling association between creatinine and uric acid was significantly steeper in males and the slope of the curve modeling association between uric acid and hsCRP was significantly steeper in females. These findings might be attributed to higher levels of serum creatinine in men and due to a higher prevalence of women in the Roma subgroup where significantly and clinically elevated levels of hsCRP were observed. In addition to the kidney function, muscle mass is believed to be the dominant determinant of the level of serum creatinine. The sex differences in serum creatinine levels observed in our study are in accordance with previously published findings of physiologically higher concentrations of creatinine in men, because they have a greater skeletal muscle mass [47]. Serum uric acid level is sex-specific, too, with men having higher levels than women. These observations may explain the observed positive associations between uric acid and serum creatinine in men in our study. The reason why we did not observe the same association in women could be due to a smaller range of values (a wider range of values tends to show a higher correlation than a smaller range). No difference in the regression curve slope was observed between sexes for blood pressure, ferritin, or BMI.

Strengths of the study include a large subject population with a high number of participants randomly selected from Roma and non-Roma, the inclusion of marginalized Roma people with high barriers to health care, and use of the wide array of clinical biochemical tests. Limitations include the cross-sectional design of the study, which precludes making inferences of causality and evaluating the outcomes longitudinally.

## 5. Conclusions

To conclude, our study has demonstrated that serum uric acid is sex- and ethnicity-specific. The comprehensive data analyses revealed considerably elevated levels of hsCRP and serum ferritin particularly in Roma males, which can reflect low-grade systemic inflammation, and thus serve as a marker of an increased cardiovascular risk. The significantly lower levels of serum uric acid along with positive linear associations with albumin may reflect the decreased antioxidant status of the Roma population. The results further support the idea that the Roma are at increased risk for cardiovascular and metabolic diseases. The observations presented here highlight the need to identify cardiometabolic disturbances in the Roma as early as possible so that timely therapeutic modification can occur. This is particularly important for the management of less-responsive patients with comorbidities.

## Figures and Tables

**Table 1:** Summary of the study parameters by sex and ethnicity.

	Roma Males (*n* = 159)		Non-Roma Males (*n* = 185)			Roma Females (*n* = 293)		Non-Roma Females (*n* = 218)		
	Mean	Standard Deviation	Mean	Standard Deviation	*p*	Mean	Standard Deviation	Mean	Standard Deviation	*p*
Age (years)	33.78	9.25	33.03	7.41	0.414	35.16	9.06	33.92	7.40	0.095
BMI (kg/m^2^)	26.79	5.94	25.79	4.26	0.079	26.44	6.01	24.07	4.36	<0.0001
Systolic blood pressure (mmHg)	127	16	125	14	0.218	120	19	118	15	0.322
Diastolic blood pressure (mmHg)	77	12	77	10	0.861	74	11	75	10	0.074
Uric acid (umol/L)	265.7	89	304.65	84.62	<0.0001	205.06	65.07	220.11	62.08	0.009
Albumin (mg/L)	47.68	2.57	47.77	2.57	0.747	46.01	2.69	46.61	3.28	0.024
Cystatin C (mg/L)	0.64	0.14	0.64	0.18	0.976	0.58	0.17	0.55	0.16	0.034
Creatinine (umol/L)	92	9.58	91.70	11.06	0.788	76.36	7.78	79.18	7.09	<0.0001
AST (ukat/L)	0.39	0.55	0.37	0.20	0.669	0.26	0.20	0.29	0.21	0.095
ALT (ukat/L)	0.32	0.49	0.33	0.22	0.788	0.19	0.19	0.19	0.13	0.762
GMT (ukat/L)	0.65	1.18	0.65	0.74	0.981	0.31	0.47	0.34	0.49	0.470
Fe (mmol/L)	18.03	7.16	19.32	6.55	0.081	14.57	6.03	17.90	7.22	<0.0001
Feritin (mg/L)	403.9	391.84	302.67	236.26	0.004	102.66	107.05	71.00	93.81	<0.0001
hsCRP (mg/L)	3.30	4.08	1.72	2.67	<0.0001	2.95	3.96	2.19	2.95	0.014
Poverty (%)	52.2		11.4		<0.0001	46.1		12.8		<0.0001
Alcohol daily > 20 g (%)	15.7		17.5		0.8	3.1		5.7		0.319
Smokers (%)	62.9		34.6		<0.0001	58		22.6		<0.0001

*n* = total number of participants in each group, patients excluded based on data availability for individual analyses. BMI—body mass index; AST—aspartate-aminotransferase; ALT—alanine-aminotransferase; GMT—gamma-glutamyl transferase; Fe—serum iron level; hsCRP—high sensitivity C-reactive protein.

**Table 2:** Relationship between uric acid adjusted for age and sex and dependent variables in all study participants.

Dependent Variable	*n*	Mean	Standard Deviation	B (linreg) × 10 Unstandardized	Std. Error of B	Beta Stand.	*p*
Age (years)	838	34.13	8.39	0.102	0.039	0.117	0.008
BMI (kg/m^2^)	828	25.76	5.35	0.202	0.023	0.317	<0.0001
Systolic blood pressure (mmHg)	826	122	17	0.249	0.072	0.147	0.001
Diastolic blood pressure (mmHg)	826	75	11	0.19	0.047	0.127	<0.0001
Uric acid (umol/L)	847	241.96	83.62	predictor	predictor	N/A	N/A
Albumin (mg/L)	847	46.86	2.90	0.036	0.013	0.104	0.005
Cystatin C (mg/L)	847	0.60	0.17	0.002	0.001	0.112	0.003
Creatinine (umol/L)	847	83.35	11.28	0.326	0.04	0.242	<0.0001
AST (ukat/L)	847	0.32	0.30	0.004	0.001	0.102	0.008
ALT (ukat/L)	846	0.24	0.28	0.002	0.001	0.074	0.052
GMT (ukat/L)	847	0.46	0.74	0.01	0.003	0.118	0.002
Fe (mmol/L)	847	17.10	6.93	0.07	0.031	0.084	0.027
Feritin (mg/L)	847	194.44	253.50	4.413	0.998	0.145	<0.0001
hsCRP (mg/L)	847	2.55	3.53	0.09	0.016	0.213	<0.0001
Poverty				−0.805	0.563	−0.45	0.153
Alcohol daily > 20 g				2.44	0.905	0.085	0.007
Smokers				−2.096	0.519	−0.125	<0.0001

Column 1 shows all dependent variables; uric acid, adjusted for age and sex, is the only predictor in all analyses. BMI—body mass index; AST—aspartate-aminotransferase; ALT—alanine-aminotransferase; GMT—gamma-glutamyl transferase; Fe—serum iron level; hsCRP—high sensitivity C-reactive protein; Std. error—standard error.

**Table 3:** Relationship between uric acid adjusted for age and sex and dependent variables in the Roma subgroup.

Dependent Variable	*n*	Mean	Standard Deviation	B (Linreg) × 10 Unstandardized	Std. Error of B	Beta Stand.	*p*
Age (years)	447	34.67	9.14	0.167	0.058	0.145	0.005
BMI (kg/m^2^)	435	36.57	5.98	0.289	0.036	0.385	<0.0001
Systolic blood pressure (mmHg)	434	123	18	0.259	0.109	0.116	0.018
Diastolic blood pressure (mmHg)	434	75	12	0.213	0.07	0.145	0.003
Uric acid (umol/L)	446	226.54	79.80	predictor	predictor	N/A	N/A
Albumin (mg/L)	446	46.60	2.77	0.046	0.017	0.133	0.007
Cystatin C (mg/L)	446	0.60	0.16	0.002	0.001	0.104	0.04
Creatinine (umol/L)	446	81.90	11.29	0.221	0.053	0.155	<0.0001
AST (ukat/L)	446	0.31	0.37	0.003	0.002	0.068	0.185
ALT (ukat/L)	445	0.24	0.34	0.001	0.002	0.031	0.540
GMT (ukat/L)	446	0.43	0.81	0.004	0.005	0.038	0.458
Fe (mmol/L)	446	15.80	6.65	0.049	0.042	0.059	0.243
Feritin (mg/L)	446	209.33	286.97	6.058	1.567	0.167	<0.0001
hsCRP (mg/L)	446	3.07	4.00	0.125	0.025	0.249	<0.0001
Poverty				0.903	0.702	0.059	0.187
Alcohol daily > 20 g				1.155	1.341	0.039	0.389
Smokers				−1.951	0.717	−1.21	0.007

BMI—body mass index; AST—aspartate-aminotransferase; ALT—alanine-aminotransferase; GMT—gamma-glutamyl transferase; Fe—serum iron level; hsCRP—high sensitivity C-reactive protein; Std. error – standard error.

**Table 4:** Relationship between uric acid adjusted for age and sex and dependent variables in the non-Roma subgroup.

Dependent Variable	*n*	Mean	Standard Deviation	B (linreg) × 10 Unstandardized	Std. Error of B	Beta Stand.	*p*
Age (years)	391	33.51	7.41	0.062	0.051	0.071	0.230
BMI (kg/m^2^)	393	24.87	4.39	0.164	0.027	0.319	<0.0001
Systolic blood pressure (mmHg)	392	122	15	0.283	0.097	0.162	0.004
Diastolic blood pressure (mmHg)	392	76	10	0.142	0.065	0.123	0.031
Uric acid (umol/L)	401	259.11	84.53	predictor	predictor	N/A	N/A
Albumin (mg/L)	401	47.15	3.03	0.021	0.02	0.059	0.298
Cystatin C (mg/L)	401	0.59	0.17	0.003	0.001	0.137	0.016
Creatinine (umol/L)	401	84.95	11.06	0.44	0.06	0.337	<0.0001
AST (ukat/L)	401	0.33	0.21	0.005	0.001	0.190	0.001
ALT (ukat/L)	401	0.25	0.19	0.004	0.001	0.175	0.001
GMT (ukat/L)	401	0.48	0.64	0.018	0.004	0.246	<0.0001
Fe (mmol/L)	401	18.56	6.94	0.049	0.048	0.06	0.308
Feritin (mg/L)	401	177.88	209.23	4.745	1.19	0.191	<0.0001
hsCRP (mg/L)	401	1.98	2.83	0.09	0.019	0.266	<0.0001
Poverty				−0.821	1.142	0.032	0.473
Alcohol daily > 20 g				3.127	1.189	0.117	0.009
Smokers				−0.846	0.829	−0.045	0.309

BMI—body mass index; AST—aspartate-aminotransferase; ALT—alanine-aminotransferase; GMT—gamma-glutamyl transferase; Fe—serum iron level; hsCRP—high sensitivity C-reactive protein; Std. error—standard error.

**Table 5:** The comparison of regression curves for variables significantly associated with uric acid in the Roma and non-Roma subgroups.

Dependent Variable	Regression Curve Roma	Regression Curve Non-Roma	*p*-Constants	*p*-Coefficients
Systolic blood pressure (mmHg)	105.361 + 0.259 × (UA/10)	106.884 + 0.283 × (UA/10)	0.05	0.129
Diastolic blood pressure (mmHg)	57.483 + 0.213 × (UA/10)	63.019 + 0.142 × (UA/10)	0.833	0.747
Cystatin C (mg/L)	0.554 + 0.002 × (UA/10)	0.598 + 0.003 × (UA/10)	0.128	0.383
Creatinine (umol/L)	95.296 + 0.221 × (UA/10)	89.196 + 0.44 × (UA/10)	0.812	0.475
Feritin (mg/L)	324.635 + 6.058 × (UA/10)	263.04 + 4.745 × (UA/10)	0.765	0.054
hsCRP (mg/L)	−2.234 + 0.125 × (UA/10)	−4.221 + 0.061 × (UA/10)	0.845	0.042
BMI (kg/m^2^)	13.897 + 0.289 × (UA/10)	15.609 + 0.164 × (UA/10)	<0.0001	<0.0001

Regression formulas are shown from age and sex adjusted model (age and sex regression coefficients are omitted). UA—uric acid; hsCRP—high-sensitivity C-reactive protein; BMI—body mass index.

**Table 6:** Relationship between uric acid adjusted for age and sex and dependent variables in males.

Dependent Variable	*n*	Mean	Standard Deviation	B (linreg) × 10 Unstandardized	Std. Error of B	Beta Stand.	*p*
Age (years)	341	33.38	8.32	0.149	0.052	0.159	0.004
BMI (kg/m^2^)	340	26.25	5.12	0.178	0.03	0.311	<0.0001
Systolic blood pressure (mmHg)	341	126	15	0.298	0.093	0.176	0.001
Diastolic blood pressure (mmHg)	341	77	11	0.152	0.066	0.123	0.021
Uric acid (umol/L)	343	286.70	88.70	predictor	predictor	N/A	N/A
Albumin (mg/L)	343	47.73	2.57	0.048	0.016	0.165	0.003
Cystatin C (mg/L)	343	0.64	0.16	0.003	0.001	0.165	0.003
Creatinine (umol/L)	343	91.84	10.39	0.454	0.062	0.388	<0.0001
AST (ukat/L)	343	0.38	0.40	0.004	0.003	0.079	0.162
ALT (ukat/L)	342	0.32	0.37	0.003	0.002	0.073	0.201
GMT (ukat/L)	343	0.65	0.96	0.01	0.006	0.066	0.094
Fe (mmol/L)	343	18.73	6.86	0.103	0.043	0.133	0.017
Feritin (mg/L)	343	349.25	321.03	6.57	1.939	0.181	0.001
hsCRP (mg/L)	343	2.45	3.48	0.065	0.021	0.164	0.002
Poverty				1.748	1.129	0.091	0.123
Alcohol daily > 20 g				2.058	1.239	0.087	0.098
Smokers				−1.298	0.970	−0.073	0.182

BMI—body mass index; AST—aspartate-aminotransferase; ALT—alanine-aminotransferase; GMT—gamma-glutamyl transferase; Fe—serum iron level; hsCRP—high sensitivity C-reactive protein; Std. error—standard error.

**Table 7:** Relationship between uric acid adjusted for age and sex and dependent variables in females.

Dependent Variable	*n*	Mean	Standard Deviation	B (linreg) × 10 Unstandardized	Std. Error of B	Beta Stand.	*p*
Age (years)	497	34.64	8.42	0.076	0.06	0.058	0.203
BMI (kg/m^2^)	488	25.42	5.49	0.295	0.036	0.340	<0.0001
Systolic blood pressure (mmHg)	485	119	17	0.276	0.114	0.104	0.016
Diastolic blood pressure (mmHg)	485	74	11	0.219	0.072	0.131	0.003
Uric acid (umol/L)	504	211.51	64.18	predictor	predictor	N/A	N/A
Albumin (mg/L)	504	46.27	2.97	0.02	0.021	0.043	0.342
Cystatin C (mg/L)	504	0.57	0.16	0.002	0.001	0.077	0.088
Creatinine (umol/L)	504	77.57	7.61	0.176	0.053	0.146	0.001
AST (ukat/L)	504	0.27	0.21	0.005	0.001	0.143	0.002
ALT (ukat/L)	504	0.19	0.17	0.002	0.001	0.083	0.068
GMT (ukat/L)	504	0.32	0.48	0.011	0.003	0.147	0.001
Fe (mmol/L)	504	16.00	6.76	−0.009	0.047	−0.008	0.853
Feritin (mg/L)	504	89.09	102.69	3.538	0.708	0.217	<0.0001
hsCRP (mg/L)	504	2.62	3.58	0.046	0.018	0.108	<0.012
Poverty				−0.307	0.659	−0.023	0.641
Alcohol daily > 20 g				2.630	1.410	0.084	0.063
Smokers				−1.459	0.610	−0.115	0.017

BMI—body mass index; AST—aspartate-aminotransferase; ALT—alanine-aminotransferase; GMT—gamma-glutamyl transferase; Fe—serum iron level; hsCRP—high-sensitivity C-reactive protein; Std. error—standard error.

**Table 8:** The comparison of regression curves for variables significantly associated with uric acid in males and females.

Dependent Variable	Regression Curve Males	Regression Curve Females	*p*-Constants	*p*-Coefficients
Systolic blood pressure (mmHg)	111.474 + 0.298 × (UA/10)	89.534 + 0.276 × (UA/10)	0.071	0.752
Diastolic blood pressure (mmHg)	58.378 + 0.152 × (UA/10)	52.653 + 0.219 × (UA/10)	0.132	0.352
Creatinine (umol/L)	83.069 + 0.454 × (UA/10)	65.564 + 0.176 × (UA/10)	0.001	0.01
Feritin (mg/L)	68.742 + 6.57 × (UA/10)	−0.227 + 3.538 × (UA/10)	0.001	0.172
hsCRP (mg/L)	0.638 + 0.065 × (UA/10)	−1.108 + 0.162 × (UA/10)	0.029	0.001
BMI (kg/m^2^)	18.637 + 0.178 × (UA/10)	18.521 + 0.295 × (UA/10)	<0.0001	0.062

Regression formulas are shown from age and sex-adjusted model (age and ethnicity regression coefficients are omitted). UA—uric acid; hsCRP—high sensitivity C-reactive protein; BMI—body mass index.

## Supplementary Materials

The following are available online at https://www.mdpi.com/1660-4601/17/20/7673/s1, Table S1: Relationship between uric acid adjusted for age, sex and BMI and dependent variables in all study participants.

## Appendix A

HepaMeta Team members: Peter Jarcuska, Andrea Madarasova Geckova, Mária Marekova, Daniel Pella, Leonard Siegfried, Pavol Jarcuska, Lydia Pastvova, Ján Fedacko, Jana Kollarova, Peter Kolarcik, Daniela Bobakova, Zuzana Dankulincova Veselska, Ingrid Babinska, Sylvia Drazilova, Jaroslav Rosenberger, Ivan Schreter, Pavol Kristian, Eduard Veseliny, Martin Janicko, Ladislav Virag, Anna Birkova, Marta Kmetova, Monika Halanová, Darina Petrasova, Katarína Carikova, Viera Lovayova, Lucia Merkovska, Lucia Jedlickova, and Ivana Valkova.
