# Supplementary material for: Roma Ethnicity and Sex-Specific Associations of Serum Uric Acid with Cardiometabolic and Hepatorenal Health Factors in Eastern Slovakian Population: The HepaMeta Study
Source: Int J Environ Res Public Health. 2020 Oct 21;17(20):7673. doi: 10.3390/ijerph17207673 (PMC7589897; doi:10.3390/ijerph17207673)
Supplement: Supplementary file 1 [file ijerph-17-07673-s001.pdf]

Table 1. Relationship between uric acid adjusted for age, sex and BMI and dependent variables in all study participants.

Dependent variable	B (linreg) x10 unstandardized	Std. Error of B	Beta Stand.	*p*
Systolic blood pressure (mmHg)	0.021	0.017	0.041	0.217
Diastolic blood pressure (mmHg)	0.045	0.026	0.059	0.08
Albumin (mg/L)	0.312	0.091	0.107	0.001
Cystatin C (mg/L)	3.156	1.564	0.062	0.044
Creatinine (umol/L)	0.206	0.028	0.277	<0.0001
AST (ukat/L)	2.412	0.821	0.089	0.003
ALT (ukat/L)	1.265	0.917	0.042	0.168
GMT (ukat/L)	0.907	0.347	0.08	0.009
Fe (mmol/L)	0.143	0.037	0.118	<0.0001
Feritin (mg/L)	0.004	0.001	0.116	0.001
hsCRP (mg/L)	0.174	0.078	0.074	0.026
Poverty	-1.060	0.545	-0.059	0.052
Alcohol daily >20g	2.62	0.867	0.092	0.003
Smokers	-1.853	0.503	-0.11	<0.0001

BMI—body mass index; AST—aspartate-aminotransferase; ALT—alanine-aminotransferase; GMT—gamma-glutamyl transferase; Fe—serum iron level; hsCRP—high sensitivity C-reactive protein.
